# Serum bisphenol A levels in girls with central precocious puberty

**DOI:** 10.1186/1687-9856-2013-S1-P71

**Published:** 2013-10-03

**Authors:** Shin Hye Kim, Mi Jung Park, Duk Hee Kim

**Affiliations:** 1 Department of Pediatrics, Sanggye Paik Hospital, Inje University College of Medicine, Seoul, Korea; 2 Department of Pediatrics, Sowha children's hospital, Seoul, Korea

## 

Bisphenol A (BPA) is a chemical widely used to make polycarbonate plastics and epoxy resins lining food and beverage containers. A number of in vitro and in vivo studies have demonstrated that BPA has an estrogenic effect by binding to the nuclear estrogen receptor, and early BPA exposure could in induce early puberty. However, effects of human exposure to BPA on pubertal onset and the association of gonadotropin levels have not been fully evaluated. We aimed to study whether serum bisphenol A levels are associated with central precocious puberty (CPP) in Korean children.

A total of 103 girls (51 CPP cases and 52 controls, aged 7 to 9 yr) were enrolled. Pubertal staging, anthropometry, bone maturation were assessed. Gonadotropin releasing hormone-stimulation test were conducted to determine the basal and peak levels of luteinizing hormone (LH). Serum bisphenol A levels were analysed by gas chromatography/mass spectrometry method. Geometric mean serum BPA levels were higher in CPP girls than in controls (6.5±5.9 vs. 3.4±4.1 ng/mL, P<0.0001). In partial correlation analysis controlling for age and body mass index, serum BPA level showed significant positive correlation with bone age (r=0.343, P=0.001), fat mass (r=0.241, P=0.021), waist circumference (r=0.223, P=0.034), basal LH levels (r=0.243, P=0.02), and peak LH levels (r=0.294, P=0.005). Bone age, height, basal/peak LH levels and prevalence of CPP increased significantly with increasing tertile of serum BPA. Increased risk of CPP [Odds ratio(95% Confidence Interval)] was observed across increasing serum BPA tertile [2.61(0.86-7.93) for tertile 2, 7.68(2.34-25.19) for tertile 3] after adjusting for age and BMI.

Serum BPA level was higher in CPP girls compared with controls, and higher serum BPA level was associated with increased risk of CPP. Prospective studies are needed to determine potential causal links between BPA exposure and CPP.

**Table:** Multivariate logistic regression analysis for obesity according to the quartiles of BPA concentrations

BPA Tertile^†^	Crude OR (95% CI)	P-value	Adjusted OR^*^ (95% CI)	P-value
1 (ref)	1.0		1.0	
2	2.02(0.75-5.46)	0.165	2.60(0.858-7.93)	0.091
3	6.67(2.31-19.25)	<0.0001	7.68(2.34-25.19)	0.001

^*^Adjusted for age and body mass index

^†^Tertile 1, 0.19~3.82ng/mL; tertile 2, 3.83~8.78 ng/mL; tertile 3, 8.79~38.1 ng/Ml

